# Plant polysaccharides with anti-lung injury effects as a potential therapeutic strategy for COVID-19

**DOI:** 10.3389/fphar.2022.982893

**Published:** 2022-10-20

**Authors:** Peng Huang, Jiahui Zhang, Wenqian Duan, Juying Jiao, Aijing Leng, Jialin Qu

**Affiliations:** ^1^ Clinical Laboratory of Integrative Medicine, The First Affiliated Hospital of Dalian Medical University, Dalian, China; ^2^ Department of Respiratory Medicine, Suixian People’s Hospital of Henan, Shangqiu, China; ^3^ Shanghai Medical College, Fudan University, Shanghai, China; ^4^ Department of Traditional Chinese Medicine, The First Affiliated Hospital of Dalian Medical University, Dalian, China

**Keywords:** lung injury, plant polysaccharides, traditional Chinese medicine, botanical drugs, coronavirus disease 2019

## Abstract

When coronavirus disease 2019 (COVID-19) develops into the severe phase, lung injury, acute respiratory distress syndrome, and/or respiratory failure could develop within a few days. As a result of pulmonary tissue injury, pathomorphological changes usually present endothelial dysfunction, inflammatory cell infiltration of the lung interstitium, defective gas exchange, and wall leakage. Consequently, COVID-19 may progress to tremendous lung injury, ongoing lung failure, and death. Exploring the treatment drugs has important implications. Recently, the application of traditional Chinese medicine had better performance in reducing fatalities, relieving symptoms, and curtailing hospitalization. Through constant research and study, plant polysaccharides may emerge as a crucial resource against lung injury with high potency and low side effects. However, the absence of a comprehensive understanding of lung-protective mechanisms impedes further investigation of polysaccharides. In the present article, a comprehensive review of research into plant polysaccharides in the past 5 years was performed. In total, 30 types of polysaccharides from 19 kinds of plants have shown lung-protective effects through the pathological processes of inflammation, oxidative stress, apoptosis, autophagy, epithelial–mesenchymal transition, and immunomodulation by mediating mucin and aquaporins, macrophage, endoplasmic reticulum stress, neutrophil, TGF-β1 pathways, Nrf2 pathway, and other mechanisms. Moreover, the deficiencies of the current studies and the future research direction are also tentatively discussed. This research provides a comprehensive perspective for better understanding the mechanism and development of polysaccharides against lung injury for the treatment of COVID-19.

## Graphical Abstract

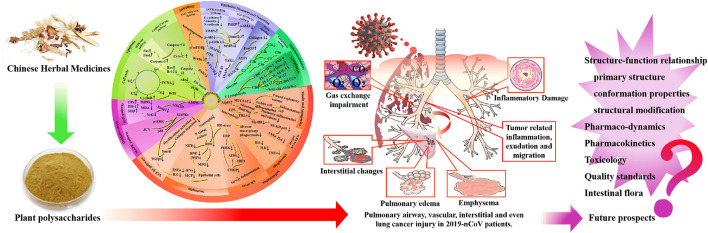

## 1 Introduction

Severe acute respiratory syndrome–coronavirus 2 (SARS-CoV-2) was detected in Wuhan, Hubei Province, China, in December 2019, resulting in the pneumonia disease named coronavirus disease 2019 (COVID-19, also named 2019-nCoV). Person-to-person transmission of COVID-19 through droplets and short-distance contact boosts the rate of infection. Millions of people have been infected in this short period of time worldwide (Fwca et al., 2020). Transmission electron microscopy images show that coronaviruses are spherical-shaped viruses with spike proteins projecting from the virion surface, making them resemble solar crowns (Ashour et al., 2020). Humans can be infected by various subtypes of coronaviruses, such as alpha, beta, gamma, and omicron (Anka et al., 2021).

COVID-19 is likely to infect the lower respiratory tract, leading to acute lung injury (ALI)/acute respiratory distress syndrome (ARDS), septic shock, and multi-organ failure, with a high case fatality ratio (Tian et al., 2020). It mainly induces lung injury *via* exploiting the angiotensin-converting enzyme II (ACE2) receptor for receptor-mediated endocytosis into the lung alveolar epithelial cells of the host (Shang et al., 2020). The commonest chest radiological performance of COVID-19 patients is multifocal or mixed ground-glass opacities or paving pattern in some cases, followed by consolidation, irregular interlobular septal thickening, and air bronchogram (Maity et al., 2019). Pleural effusion is rare or only occurs in severe COVID-19 patients. The major pathological manifestations include bilateral diffuse alveolar damage, interstitial inflammation, and fibrosis (Bakowitz et al., 2012). Furthermore, pleural lesions, mucous plugs, and inflammatory cell infiltration were also noticed (Bhatia and Moochhala, 2004; Rubenfeld and Herridge, 2007). Some vaccines or drugs are approved to fight against COVID-19. However, the clinical utility of which is limited.

**FIGURE 1:**
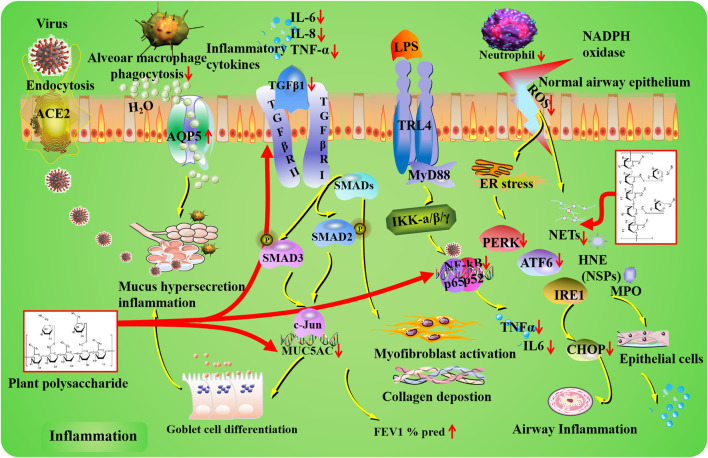
Inflammation mechanisms of plant polysaccharides against lung injury. COVID-19 induces lung inflammation *via* ACE2 (angiotensin-converting enzyme II) receptor-mediated endocytosis into the airway epithelium. Plant polysaccharides have potential regulatory effects on MUC5AC expression, goblet cell differentiation, mucus secretion, and expiratory volume in 1 s percent predicted (FEV1% pred). In the TLR4/NF-κB signaling pathway, inflammatory cytokines can be affected by plant polysaccharides *via* targeting NF-κB. Plant polysaccharides could improve airway inflammation through the modulation of PERK, ATF6, and CHOP. Plant polysaccharides could decrease neutrophil levels and restrain NET formation. The TGF-β1 pathway can be regulated by plant polysaccharide.

**FIGURE 2:**
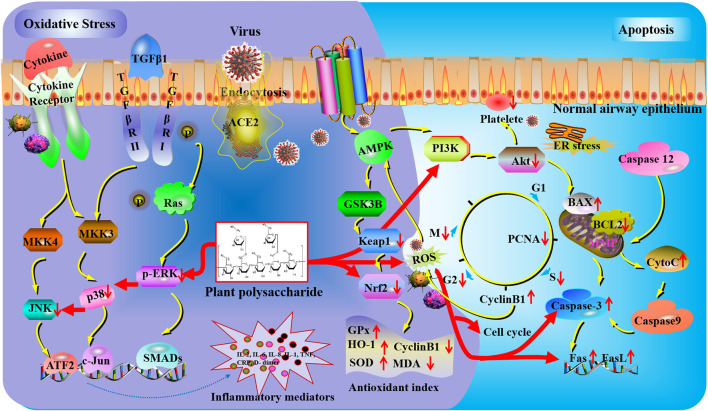
Oxidative stress and apoptosis mechanisms of plant polysaccharides against lung injury. COVID-19 induces lung oxidative stress injury *via* ACE2 (angiotensin-converting enzyme II) receptor-mediated endocytosis into the airway epithelium. Plant polysaccharides could affect reactive oxygen species (ROS) generation and nuclear factor erythroid 2-related factor 2 (Nrf2) activation to regulate oxidative damage. The expression of c-Jun N-terminal kinases (JNKs), phosphorylated extracellular signal-regulated kinase (p-ERK), and p38 were obviously inhibited by plant polysaccharides. The activation of platelets could be suppressed by plant polysaccharides *via* the PI3K/AKT pathway. Plant polysaccharides can affect caspase-3 to regulate the gene expression of fatty acid synthase (Fas) and Fas antigen ligand (FasL).

**FIGURE 3:**
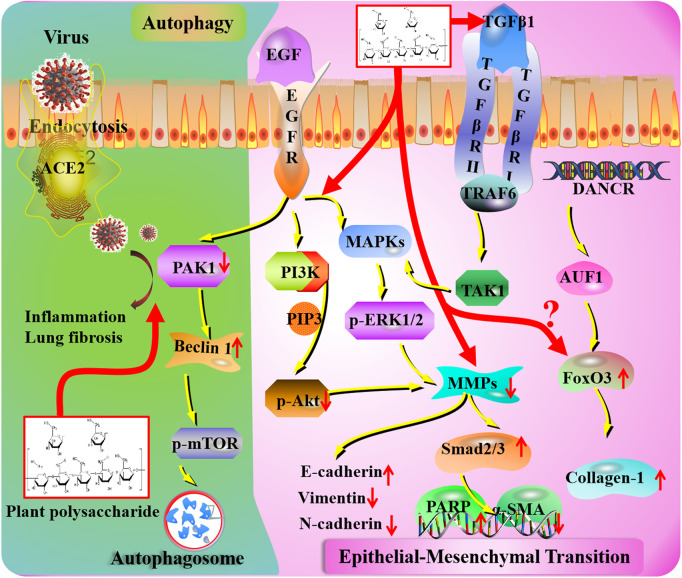
Autophagy and epithelial–mesenchymal transition mechanisms of plant polysaccharides against lung injury. COVID-19 induces lung inflammation and fibrosis *via* ACE2 (angiotensin-converting enzyme II) receptor-mediated endocytosis into the airway epithelium. The ACE2-mediated PAK1 (p21-activated kinase) signaling pathway is a potential therapeutic target of plant polysaccharides. Plant polysaccharides affect MMP (metalloproteinase) levels in COVID-19 patients. Plant polysaccharides could act on MAPKs (mitogen-activated protein kinases), PI3K (phosphatidylinositide 3-kinase), and TGF-β1 (transforming growth factor beta) to stimulate fibroblast proliferation. Unknown links exist between COVID-19 and FoxO (forkhead box) transcription factor.

**FIGURE 4:**
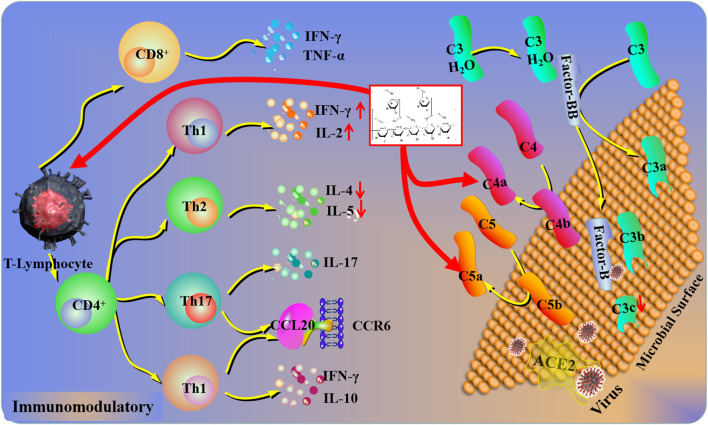
Immunomodulatory mechanisms of plant polysaccharides against lung injury. COVID-19 induces a pulmonary immune response *via* ACE2 (Angiotensin-converting enzyme II) receptor-mediated endocytosis into the airway epithelium. In response to COVID-19, the complement system in lung epithelial cells could be activated, which was driven by the transcription of factor B and C3a. Plant polysaccharides can affect the levels of C5a, C4a, and C3b. In addition, the lymphocyte activation could be inhibited by plant polysaccharides *via* the complement system.

China is sitting on a gold mine of well-recorded and traditionally well-practiced knowledge of herbal medicine. Several systems of traditional medicine such as traditional Chinese medicine (TCM), traditional Mongolian medicine, Tibetan medicine, Uighur medicine, and Miao medicine are being practiced for thousands of years in China. Multiple traditional Chinese medicines were applied in the treatment of flu-like symptoms, asthma, inflammation, tonsillitis, and sore throat in COVID-19 patients. Among them, Xuanfei Baidu decoction was recommended to treat mild and moderate COVID-19 patients and proved to effectively alleviate symptoms. The aqueous extract of Chinese herbal is enriched with several chemical compounds, among which polysaccharides are the most active constituents (Yu et al., 2018; Maity et al., 2019). Interestingly, medicinal plants, with rhizomes or fruits as their medicinal parts, contain plenty of polysaccharides, which are an essential part of their therapeutic effect. Low toxicity, high molecular mass, unique branching configuration, and conformation of polysaccharides may alleviate lung injury in the event of pathogenic invasion of viral diseases. The present review focuses on lung injury through regulating inflammation, oxidative stress, apoptosis, and other mechanisms of polysaccharides from plant resources to explore the possibility of the emergence of new medicine for the treatment of COVID-19. Therefore, the underlying lung protective mechanisms of plant polysaccharides are comprehensively classified and elaborated in this review (Table 1; Figures 1–4).

**TABLE 1:** Underlying protective mechanisms of plant polysaccharides against lung injury.

Compound name	Source	Medical parts	Pinyin	Monosaccharide composition^a^	M.W. (Da)	Types of lung diseases	Model	Administration involved				Involved pathway	Observation^b^	Reference index
								Route		—	Mechanism			
DOP	*Dendrobium officinale Kimura* and *Migo*	Stem	Shi Hu	ND	ND	Cigarette smoke extract-induced COPD	MLE-12 cells, SD rats, and patients	*In vivo* and *in vitro*		i.g. and po.	Inflammation	Mucin and aquaporins and inflammatory index	MUC5AC↓, AQP5↑, FEV1/FVC↑, IL-6↓, IL-8↓, TNF-α↓, and CRP↓	Song et al. (2016)
AMP	*Astragalus mongholicus Bunge*	Root	Huang Qi	ND	ND	OVA-LPS-induced asthma	Female BALB/c mice	*In vivo*		i.g.	Inflammation	ER stress	Cxcl5↓, IL8↓, Ccl20↓, IL13RA↓, IL17RA↓, CHOP↓, p-PERK↓, ATF6↓, NF-κB↓, MUC5AC↓, MUC5B↓, and IgE↓	Lu et al. (2016)
CDP	*Cedrus deodara (Roxb. ex D.Don) G.Don*	leave and twig	Xiang Bai	Man:Rha:GlcA:GalUA:Glc:Gal: Ara=2.96: 3.13: 1.05: 34.33: 4.04: 8.62: 42.63	1.5 ×10^4^ to 8.12 ×10^5^	H1N1-induced lung injury	Male BALB/C mice	*In vivo*		i.g.	Inflammation, oxidative stress, and immunomodulatory	Inflammatory index, antioxidant index, and complement system	NF-κB p65↓, C3c↓, MDA↓, MPO↓, and SOD↑	Fu et al. (2019)
CDP	*Cedrus deodara (Roxb. ex D.Don) G.Don*	Leave and twig	Xiang Bai	GalA:Gal:Ara=0.26: 0.14: 0.58	1.89×10^5^	H1N1-induced lung injury	Male BALB/C mice	*In vivo*		i.g.	Inflammation, oxidative stress, and immunomodulatory	Inflammatory index, antioxidant index, and complement system	NF-κB p65↓, MDA↓, MPO↓, SOD↑, and C3c↓	Fu et al. (2019)
NTP	*Nitraria tangutorum Bobrov*	Fruit	Bai Ci	ND	ND lung injury	LPS induced	C57BL/6 mice	*In vivo*		i.g.	Inflammation, oxidative stress, and antioxidant index	TLR4/NF-κB pathway TNF-α↓, IL-1β↓, and IL-6↓	TLR4↓, pIKKα/β↓, MDA↓, SOD↑, and p-NF-κB↓	Meng et al. (2021)
CDMP	*Cistanche deserticola Ma*	Rhizome	Rou Cong Rong	Man:Glc:Gal:Fuc= 1.0:5.16:4.75:1.34	ND	Bleomycin-induced fibrosis	Male Kunming mice and A549 cells	*In vivo* and *in vitro*		i.g.	Inflammation and oxidative stress	Antioxidant index, TLR4/NF-κB pathway, and MAPK pathway	TNF-α↓, IL-6↓, IL-1β↓, SOD↑, MDA↓, GSH-Px↑, TLR4↓, MyD88↓, NF-κB p-p65/NF-κB p65↓, ROS↓, Nrf2↑, HO-1↑, p-ERk↓, p38↓, JUK↓, α-SMA↓, Smad3↓, Smad7↑, collagen I↓, and TGF-β1↓,	Dong et al. (2020)
HCP	*Houttuynia cordata Thunb.*	Dried whole plant	Yu Xing Cao	Glc:Gal:Ara:Rha= .40:2.14:1.17:1	ND	Influenza virus-induced pneumonia	Male BALB/c mice	*In vivo*		i.g.	Inflammation and immunomodulatory	TLR4-NF-κB pathway and lung–gut axis	sIgA↑, TLR4↓, and p-NFκbp65↓	Zhu et al. (2018)
AMP	*Astragalus mongholicus Bunge*	Root	Huang Qi	ND	ND	PM2.5 induced	Balb/c mice	*In vivo*		i.g.	Inflammation	Macrophage	Macrophage phagocytosis ↓	Chu et al. (2016)
CPP	*Codonopsis pilosula (Franch.) Nannf.*	—	Dang Shen	—	—	COPD	—	—		—	—	Inflammatory index	IL-6↓, IL-8↓, and TNF-α↓	—
HCP	*Houttuynia cordata Thunb.*	Dried whole plant	Yu Xing Cao	Glc:Gal:Ara:Rha= 3.40:2.14:1.17:1.00	ND	LPS-induced lung injury	Male BALB/c mice and peritoneal macrophages	*In vivo* and *in vitro*		i.g.	Inflammation and immunomodulatory	Macrophage and complement system	TLR4↓, TNF-α↓, IL-6↓, IL-1β↓, NO↓, C3d↓, and complement deposition↓	Xu et al. (2015)
BCP	*Bupleurum chinense DC.*	Root	Bei Chai Hu	Gal:Ara:Glc:Rha:Man= 13.43: 11.57: 4.02: 1.02: 1.0	ND	LPS-induced lung injury	Male Kunming mice	*In vivo*		i.g.	Inflammation and immunomodulatory	Neutrophil and complement system	MPO↓, TNF-α↓, and NO↓; inhibition of complement activation	Xie et al. (2012)
BSSP	*Bassia scoparia (L.) A.J.Scott*	Fruit	Di Fu Zi	Man:Rha:GalUA:Glc: Gal:Ara= 0.39:0.54:1.00:6.25:1.25:1.35	3×10^5^	LPS-induced lung injury	Male ICR mice	*In vivo*		i.g.	Inflammation	Neutrophil and inflammatory index	Neutrophil↓, MPO↓, TNF-α↓, and IL-6↓	Chen et al. (2017)
BSP	*Bletilla striata (Thunb.) Rchb.f.*	Rhizome	Bai Ji	ND	ND	Acute lung injury	Male ICR mice	*In vivo*		i.g.	Inflammation	Inflammatory index	IL-6↓, IFN-γ↓, TNF-α↓, and MCP-1↓	Chang et al. (2018)
DOP	*Dendrobium officinale Kimura* and *Migo*	Stem	Shi Hu	Man:Glc:Ara=5.55:1:0.12	3.938×10^5^	PM2.5-induced lung injury	Male BALB/c mice and BEAS-2B cells	*In vivo In vitro*		i.g.	Inflammation and oxidative stress	TLR4 signaling pathway, antioxidant index, and Nrf2 signaling pathway	TLR4↓, Nrf2↑, HO-1↑, NQO-1↑ ROS↓, MDA↓, SOD↑, and GSH-Px↑,	Wen et al. (2021)
AEP	*Arnebia euchroma (Royle ex Benth.) I.M.Johnst.*	Root	Zi Cao	Ara:Glc:GaL:Man:Rha= 3.69:2.57:1.47:1.20:1	ND	LPS-induced lung injury	Male SD rats	*In vivo*		i.g.	Oxidative stress and immunomodulatory	Antioxidant index and complement system	MPO↓, SOD↑, C3↓, and C4↓	Ou et al. (2017)
LBP	*Lycium barbarum L.*	Fruit	Gou Qi	ND	ND	Hyperoxic lung injury	C57BL/6 wild-type mice and pulmonary microvascular endothelial cells	*In vivo* and *in vitro*		ND	Oxidative stress	Nrf2 pathway	HO-1↑, Nrf2↑, Keap1↓, AMPK↑, IL-6↓, IL-1β↓, MDA↓, and GPx↑	Zheng et al. (2019)
GLP	*Glehnia littoralis (A.Gray) F.Schmidt ex Miq.*	Dried root and rhizome	Bei Sha Shen	ND	2.78 ×10^4^	Lung cancer	A549 cells	*In vitro*	ND		Apoptosis	Cell cycle	S and G2/M phase↓	Wu et al. (2018)
HCP	*Houttuynia cordata Thunb.*	Dried whole plant	Yu Xing Cao	ND	2.17 ×10^4^	Lung cancer	A549 cells LO2 cells	*In vitro*	ND		Apoptosis	Cell cycle	S phase↓, G1 phase↓, caspase-3↑, and cyclinB1↑	Han et al. (2018)
TFP	*Tussilago farfara. L.*	Flower buds	Kuan Dong Hua	Rha:GalUA:Glc:Gal:Ara= 13:13:1:7:12	3.78×10^4^	Lung cancer	A549 cells	*In vitro*	ND		Apoptosis	PI3K/Akt pathway	p-Akt↓, caspase-3↑, Fas↑, FasL↑, Bcl-2↓, and Bax↑	Qu et al. (2018)
SDP	*Scleromitrion diffusum (Willd.) R.J.Wang*	Dried whole plant	Bai Hua She She Cao	Glc:Gal:Man=2.0:1.0:1.0	8.9 ×10^4^	Lung cancer	Male nude mice, WI38 cells, and A549 cells	*In vivo* and *in vitro*	i.g.		Apoptosis	Caspase cascade pathway	Caspase-9 and -3 ↑, cytochrome c↑, and Bax↑	Lin et al. (2019)
EPP	*Echinacea purpurea (L.) Moench*	Flower	Zi Zhui Hua	ND	ND	LPS-induced lung injury	Male C57BL/6 mice and RAW 264.7 cells	*In vivo* and *in vitro*	i.g.		Inflammation apoptosis	Caspase cascade pathway and TLR4/NF-κB pathway.	Caspase-3↓, MPO↓, TNF-α↓, IL-6↓, IL-1β↓, TLR4↓, MyD88↓, p-IκBα↓, NF-κB↓, p-NF-κB↓, IκBα↑, Bcl-2↑, and Bax↓	Zhang et L. (2020)
CLP	*Coix lacryma-jobi var. ma-yuen (Rom.Caill.) Stapf*	Seed	Yi Yi Ren	ND	1.2×10^4^	Lung cancer	A549 cells	*In vitro*	ND		Apoptosis	Caspase cascade pathway	Caspase-9 and -3 ↑	Lu et al. (2013)
IBP	*Ipomoea batatas (L.) Lam.*	Rhizome	Gan Shu	ND	ND	Lung cancer	Male BALB/c mice and A549, H460, H1299, and MLE-12 cells	*In vivo* and *in vitro*	i.g.		Autophagy	PAK1/Akt/mTOR signaling	PAK1↓ Beclin1 and Atg5↑ PI3K↑ p-Akt↓, and p-mTOR↓,	Bu et al. (2020)
HCP	*Houttuynia cordata Thunb.*	Dried whole plant	Yu Xing Cao	Man:Rha:GlcA:GalUA:Glc:Gal: Xyl:Ara=6.54:9.30:2.84: 15.32:18.66:27.88:5.64:13.82	2.29×10^5^ 1.08×10^5^ 5.76×10^4^ 3.31×10^5^	H1N1-induced lung injury	Male BALB/C C57BL/6 mice, C57BL/6 Foxp3−/− mice, and C57BL/6 RoRγt−/− mice	*In vivo*	i.g. i.v.		Immunomodulatory	Lymphocyte	IFN-α↓ IFN-β↓, IL-17A↓, IL-10↑,CCL20↓ p-STAT3↓ p-STAT5↑, and TNF-α↓ IL-6↓	Shi et al. (2020)
SDP	*Scleromitrion diffusum (Willd.) R.J.Wang*	Dried whole plant	Bai Hua She She Cao	Glc:Gal:Man=2.0:1.0:1.0.	8.9×10^4^	Lung cancer	A549 cell line	*In vitro*	ND		Epithelial– mesenchymal transition	EGFR/Akt/ERK pathways	MMP-2↓, MMP-9↓, TIMP-1↑, TIMP-2↑, TIMP-9↑, COX-2↓, N-cadherin↓, vimentin↓, E-cadherin↑, p-EGFR↓, p-ERK1/2↓, and p-Akt↓	Lin et al. (2019)
KFP	*Diospyros kaki L.f.*	Leave	Shi Ye	ND	ND	Lung cancer	A549 and EKVX cells and MLg cells	*In vitro*	ND		Epithelial– mesenchymal transition	TGF-β1/SMAD pathway	MMP-2↑, MMP-9↑, cleaved-PARP↑, and SMAD2/3↓	Lim et al. (2020)
DOP	*Dendrobium officinale Kimura* and *Migo*	Stem	Shi Hu	ND	ND	Bleomycin-induced fibrosis	Male SD rats	*In vitro*	i.g.		Epithelial– mesenchymal transition	TGF-β1/SMAD pathway	TGFβ1↓Smad2/3, pSmad2/3, collagen I↓, and fibronectin protein expression ↓	Chen et al. (2018)
ASP	*Angelica sinensis (Oliv.) Diels*	Stem	Dang Gui	ND	ND	Bleomycin-induced fibrosis	SD rats and RLE-6TN cells	*In vivo* and *in vitro*	i.g.		Epithelial– mesenchymal transition	FoxO pathway	FOXO3↑, α-SMA↓, and E-cadherin↑	Qian et al. (2020)
HCP	*Houttuynia cordata Thunb.*	Dried whole plant	Yu Xing Cao	Glc:Gal:Ara:Rha= 3.40:2.14:1.17:1	1-5×10^6^	LPS-induced lung injury	Male SD rats, Male Wistar rats, and sheep blood cells	*In vivo* and *in vitro*	i.g.		Inflammation, oxidative stress, and immunomodulatory	Neutrophil, antioxidant index, and complement system	C3↓, C4↓, SOD↑, MDA↓, and neutrophilia↓	Lu et al. (2018)
PGP	*Panax ginseng C.A.Mey.*	Rhizome	Ren Shen	ND	ND	Lung cancer	Clinical patients	*In vivo*	po.		Immunomodulatory	Lymphocyte	INF-γ↑, IL-2↑, IL-4↓, and IL-5↓	Ma et al. (2014)
PGP	*Panax ginseng C.A.Mey.*	Fruit	Ren Shen	Gal:Glc:Rha:Ara= 6.1:2.0:1.1:3.2	1.4 × 10^5^	Lung cancer	C57BL/6 mice and mouse LLC cells	*In vivo*	i.g.		Immunomodulatory	Lymphocyte	IL-2↑, IFN-γ↑, CD4+/CD8+↑, MPO↓, and TNF-α↓	Wang et al. (2015)

^a^ Gal, galactose; Ara, arabinose; Rha, rhamnose; Glc, glucose; Man, mannose; Xyl, xylose; GalUA, galacturonic acid; Fuc, fucose; GalA, galacturonic acid; GlcA, gluconic acid.

^b^ "↓", decrease; "↑", increase.

## 2 Lung-protective mechanisms of plant polysaccharides

### 2.1 Inflammation

#### 2.1.1 Mucin and aquaporins

Among all the lung airway injuries, mucus obstruction is regarded as the crucial cause of airflow limitation and mortality (Maestrelli et al., 2001). Under normal conditions of lung physiology, mucins are usually located at epithelial mucosae for lubricating and protecting the epithelial surface (Rose et al., 2001). Unfortunately, the dysfunction of mucus generation and purgation may result in the pathological changes in respiratory diseases (Rose and Voynow, 2006). Mucin-5AC (MUC5AC) is a major gel-forming glycoprotein present in the respiratory tract epithelia that protects the mucosa from infection and chemical damage through the mucociliary system (Perez-Vilar et al., 2004). The increased expression of MUC5AC has been reported in the bronchiolar epithelium and submucosal glands, whose tendency is inversely related to forced expiratory volume in 1 s percent predicted (FEV1% pred) against chronic obstructive pulmonary disease (COPD) in clinical practice (Leikauf et al., 2002; Caramori et al., 2009). In addition, the airway mucus in COPD patients is dehydrated and adhesive followed by the produced chronic inflammation, infection, and progressive loss of lung function gradually. Moreover, related studies have revealed that the regulation of aquaporins (AQPs) may contribute to expectoration by promoting mucus secretion and sputum dilution (Lu and Zheng, 2013). AQPs are a family of transmembrane proteins that are extensively expressed in various epithelial and endothelial cells for water transport (King et al., 2004). AQP5 is distributed in airway epithelial cells, type I alveolar epithelial cells, and submucosal glands with the function of transporting (Verkman et al., 2000). Researchers have proved that AQP5 knockout mice lead to more mucus secretion in the upper respiratory tract by destroying the epithelial barrier (Song and Verkman, 2001). Furthermore, Song et al. (2016) offered evidence that oral administration of *Dendrobium officinale* Kimura and Migo [Orchidaceae; Dendrobii caulis] polysaccharides (DOPs) could ameliorate pulmonary dysfunction against cigarette smoke-induced chronic pulmonary inflammatory processes *via* significantly decreasing the levels of pro-inflammatory mediators (interleukin 6, IL-6, and C-reactive protein, CRP) and remarkably downregulating MUC5AC and upregulating AQP5 in COPD models and patients. Additionally, the lung protective effect of polysaccharides isolated from *Astragalus mongholicus* Bunge [Fabaceae; Astragali radix] (AMP) against ovalbumin–lipopolysaccharide (OVA-LPS)-sensitized and challenged pulmonary injury has been reported to interfere with goblet cell differentiation by decreasing the levels of MUC5AC and MUC5B in the bronchoalveolar lavage fluid (BALF) (Lu et al., 2016).

In recent clinical practice, research studies revealed that the small airway and alveolar wall were blocked with thick and sticky mucus in critically ill COVID-19 patients, which led to difficulty in breathing (Lu et al., 2021). Further investigation suggested that mucus is a mixture of secretion by airway and alveolar epithelial cells against viruses and inflammatory mediators (Lu et al., 2021). The clearance of airway mucus may be conducive to alleviating respiratory distress, and MUC5AC perhaps serves as a potential indicator for plant polysaccharide in the treatment of COVID-19.

#### 2.1.2 TLR4/NF-κB signaling pathway

External stimuli are also the common cause of lung injury, which activate the location of inflammatory cells in the injured area and promotes the secretion of cytokines. In the early stages of pulmonary injury, interleukin-1 beta (IL-1β), tumor necrosis factor (TNF-α), interleukin 8 (IL-8), and other inflammatory factors significantly increase in BALF. IL-1β is regarded as an important target that could be secreted by activated macrophages (Mokra and Kosutova, 2015). The toll-like receptor (TLR) family plays a significant role in pathogen recognition and inflammatory response. As an important downstream transcription factor, nuclear transcription factor-κB (NF-κB) could be activated by TLRs in many developmental processes of tissues. Moreover, NF-κB plays a key role in inflammatory regulation by promoting the transcription of cytokines (TNF-α and IL-6) (Lawrence, 2009). Among TLRs, TLR4 is regarded as a major lipopolysaccharide (LPS) receptor. When expressed on the cellular membrane, downstream mediators such as NF-κB could be activated and pro-inflammatory cytokines (TNF-α and IL-6) may be increased subsequently. Previous studies revealed that LPS may combine with TLR4, which stimulated the expression of NF-κB and then led to lung injury (Dong and Yuan, 2018). Related studies have revealed that pretreatment with *Cedrus deodara* (Roxb. ex D.Don) G. Don [Cupressaceae; Pinus deodara roxb] polysaccharides (CDPs) could inhibit the expression of NF-κB in lung tissues and contribute to the repair of lung injury (Fu et al., 2019).

In addition, the stimulation of TLR4 in turn activates the toll/IL-1 receptor (TIR) domain-containing adaptor myeloid differentiation primary response gene 88 (MYD88), which then phosphorylates and activates IL-1 receptor-associated kinase-4 (IRAK-4). Also, then, phosphorylation of IRAK-4 could activate the inhibitor of the NF-kappaB kinase (IKK) complex and contribute to the degradation of IκB (inhibitor of NF-κB) α and activation of NF-κB. Recently, Meng et al. (2021) found that pretreatment with *Nitraria tangutorum* Bobrov [Nitrariaceae; Fophorae fructus] polysaccharides (NTPs) may inhibit NF-κB activation *via* inhibiting the phosphorylation of IKK, degradation of IκB α, and phosphoactivation of NF-κB. Moreover, *Cistanche deserticola* Ma [Orobanchaceae; Cistanches herba] polysaccharides (CDMPs) and *Houttuynia cordata* Thunb. [Saururaceae; Houttuyniae herba] polysaccharides (HCPs) orally administration might alleviate inflammatory responses in mice through dramatically downregulating the protein expression of TLR4, MyD88, NF-κB phospho-p65/NF-κB p65 (Zhu et al., 2018; Dong et al., 2020).

It is worth noting that the pathogenesis of critically ill patients in COVID-19 has been related to the NF-κB signaling pathway (Pfefferle et al., 2009; Hirano and Murakami, 2020). COVID-19 could hijack NF-κB and activate the expression of inflammatory cytokines, chemokines, and inducible enzymes, and then a cytokine storm would be induced. Under such circumstances, the simultaneous inhibition of NF-κB should be considered as an attractive approach for therapeutic intervention. The ACE2 receptor acts as a binding and entry site. Upon binding of the SARS-CoV-2, the serine protease could be activated and then the viral cell can access the host cell through endocytosis. Previous research studies have demonstrated that several plant polysaccharides could regulate the TLR4/NF-κB signaling pathway. Thus, the stimulation of NF-κB and its cross-talk with cell signaling pathways may serve as an effective way in COVID-19 treatment.

#### 2.1.3 Endoplasmic reticulum stress

The endoplasmic reticulum (ER) is considered a protein-folding factory. It could not only synthesize functional proteins but also transport secretory and biological membrane proteins to the Golgi apparatus in vesicles (Gaut and Hendershot, 1993). Current researchers have demonstrated that ER stress plays an important role in the pathogenesis of lung injury (Du et al., 2020). When excessive ER stress occurs, an unfolded protein response (UPR) will be initiated by an adaptive feedback mechanism, and the functional homeostasis of ER could be recovered. Furthermore, this progress could be mediated by three protein sensors in the ER, namely, double-stranded RNA-activated protein kinase RNA-like ER kinase (PERK), inositol-requiring enzyme 1 (IRE1), and activating transcription factor 6 (ATF6) (Hetz, 2012). Related data revealed that three canonical protein sensors (IRE1α, PERK, and ATF6) and two major markers (C/EBP homologous protein, CHOP, and heavy-chain binding protein, BIP) may recognize enhanced ER stress responses (Kim et al., 2013), yet it is worth noting that polysaccharides isolated from *Astragalus mongholicus* Bunge effectively attenuate OVA-LPS-induced airway inflammation by decreasing the ER stress marker CHOP, inhibiting the nuclear translocation of ATF6 and p-PERK in inflamed lung tissues (Lu et al., 2016).

It is well documented that the replication of COVID-19 in the cytoplasm is strongly associated with increased levels of reactive oxygen species and disturbances of Ca^2+^ caused by UPR-mediated ER and its transducers. Cells infected with the COVID-19 subunit showed increased levels of ER stress associated genes. In addition, a significant phosphorylation of PERK has been revealed in SARS-infected cells (Pfefferle et al., 2009). Furthermore, stimulation of ATF6 and CHOP promoter activities *via* the accessory protein 3a of SARS may conduce to the activation of PERK (Minakshi et al., 2009). AMP is an anti-inflammatory modulator during the progress of lung injury. It could improve protective mechanisms for respiratory tract disorders. Considering the properties in terms of ER stress modulation and anti-inflammatory, we predict that the botanical drug therapy may have potential against lung injury induced by COVID-19.

#### 2.1.4 Neutrophil

Neutrophil activation, infiltration, and cytokine release are key steps in the development of the inflammatory response (Chen et al., 2016). Neutrophils that play an important role in the progress of lung injury could response to chemotactic factors released by activated macrophages, pulmonary epithelial cells, and endothelial cells and build the first line of defense in innate immunity consequently. This phenomenon could be reflected by detecting the myeloperoxidase (MPO) level on account of its predominance in neutrophils. Different doses of *Bupleurum chinense* DC [Apiaceae; Bupleuri radix] *polysaccharides* (BCPs) could improve lung injury through reducing the quantity and large influx of neutrophils into the lung, accompanied by decreased total cell counts and restrained MPO activity (Xie et al., 2012).

Once the lung is injured by inflammation, reactive oxygen species (ROS), neutrophil serine proteases (NSPs), and neutrophil extracellular traps (NETs) are generated and released by stimulated neutrophils in the blood circulation (Mantovani et al., 2011). Human neutrophil elastase (HNE) is a kind of NSP that involved in neutrophil activation during microorganism invasion. Unfortunately, the alveolar structure could be destroyed by excessive release of HNE through hydrolyzing the elastin-rich tissue. Moreover, pulmonary inflammation and neutrophil migration could also be followed by HNE mediators (Sandhaus and Turino, 2013). Previous research has revealed that HNE could accelerate neutrophil accumulation by provoking the release of pro-inflammatory cytokines including TNF-α, IL-6, interferon-γ (IFN-γ), and monocyte chemoattractant protein-1 (MCP-1) from lung epithelial cells and macrophages (Benabid et al., 2012). *Bassia scoparia* (L.) A.J.Scott [Amaranthaceae; Kochiae fructus] polysaccharide (BSSP) treatment *via* oral gavage could dramatically alleviate neutrophil infiltration and reduce MPO and neutrophil elastase activity in tissue. Meanwhile, the levels of pro-inflammatory cytokines TNF-α and IL-6 in BALF were also significantly suppressed, and the variation tendency of transcription factors expression was consistent with the changes of inflammatory response (Chen et al., 2017). Moreover, polysaccharides isolated from *Bletilla striata* (Thunb.) Rchb. f [Orchidaceae; Bletillae rhizoma] (BSP) against PM2.5-induced acute lung injury have been reported to interfere the inflammatory response *via* effectively inhibiting the expression of inflammatory cytokines IL-6, IFN-γ, TNF-α, and MCP-1 (Chang et al., 2018).

ROS are generated *via* the nicotinamide adenine dinucleotide phosphate (NADPH) oxidase and nitric oxide synthase pathways. NETs refer to the net-like structures of nuclear DNA decorated with granular proteins such as HNE and MPO, which require the production of ROS and bind pathogens (Papayannopoulos et al., 2010). Once neutrophil activation occurs, HNE will translocate to the nucleus, cleave histones, stimulate chromatin decondensation, and then lead to cell rupture and NET release. Previous studies have indicated that NETs triggered lung injury in a mice model, and the formation of NETs could be restrained by HNE inhibitors (Caudrillier et al., 2012; Liu et al., 2016). In a LPS-induced ALI mice model, the NET formation was significantly increased, and this feature was suppressed by treatment with BSSP in a dose-dependent manner. This tendency consolidated the lung protective effects of BSSP in inflammatory responses (Chen et al., 2017).

Recently, the elevated level of neutrophils is an early indicator of COVID-19 infection (Veras et al., 2020) and may cause serious respiratory problems (Ma et al., 2020). Infection by COVID-19 agitates the inflammasome in monocytes and macrophages, resulting in the cytokine storm in COVID-19 patients. As the most abundant leukocytes, neutrophils could release NETs and be involved in the pathogenesis of COVID-19. The pathogenic role of NETs has been proven in various conditions including ischemia-reperfusion injury, organ fibrosis, and ALI (Sorvillo et al., 2019). In animal models of severe ARDS, NETs are enormously damaging, interfering with the blood circulation and oxygenation of the lung. The accumulation of neutrophils and NETs may be conducive to the increase in mucous viscosity, which in turn causes lung ventilatory function impairment. Interestingly, NETs may be regulated by BSSP and play a significant role against lung injury in COVID-19 patients.

#### 2.1.5 TGF-β1 pathway

At present, the etiology and pathogenesis of pulmonary fibrosis remain poorly understood, and various kinds of conditions and risk factors are weakly associated with the disease (Oikonomou et al., 2006). Previous evidence has demonstrated that transforming growth factor beta (TGF-β) plays a regulatory role in airway remodeling including mucus hyper-secretion (Zhang et al., 2018). In addition, long-term studies have shown that the important aspect of chronic inflammatory cells in the initiation and progression of fibrotic response (Keane et al., 2006). Profibrotic protein TGF-β could be activated by inflammatory cytokines (IL-1β, IL-6, and TNF-α) (Bringardner et al., 2008), and the activation of TGF-β1/Smad signaling pathways then leads to myofibroblast activation and collagen deposition (McKleroy et al., 2013). Pretreatment with CDMP protected mice pulmonary fibrosis *via* significantly downregulating the secretion of TGF-β1 along with the decrease of IL-1β, IL-6, and TNF-α levels (Dong et al., 2020).

Pulmonary fibrosis is considered a well-recognized sequela to COVID-19. In the early stages of infection, the mRNA level of TGF-β1 from upper airway samples is lower than that of controls (Montalvo Villalba et al., 2020). Recently, Maranatha et al. (2022) reported that serum TGF-β1 levels in severe and critical COVID-19 were lower than non-severe COVID-19. When the alveolar epithelial cells were damaged, TGF-β and TNF-α were secreted. These mediators can facilitate the formation of fibroblasts and then lead to collagen deposition. Research on COVID-19 differentially expressed genes revealed that the TNF pathway is one of the mechanisms of pulmonary fibrosis secondary to COVID-19 (Yu et al., 2021). Therefore, the regulation of TGF-β1 target may be a therapeutic method *via* applying plant polysaccharide in clinical.

### 2.2 Oxidative stress

#### 2.2.1 Antioxidant index

The lung that evolved as a susceptible organ could efficiently facilitate gas exchange for physiological activity. Oxygen could not only satisfy the basic need but also transformed into ROS and reactive nitrogen species through enzymatic and non-enzymatic processes and lead to protein, lipid, and DNA damage (Domej et al., 2014). It is well known that oxidant production could result in the development and progression of lung injury, which always leads to irreversible interstitial changes (Kinnula et al., 2005). Extrinsic oxidants such as paraquat could be metabolized into free radicals after inhaling, leading to pulmonary fibrosis (Bus et al., 1976). Intrinsically generated oxidant was usually derived from the mitochondria. Nevertheless, phagocytic cells (residential macrophages and recruited neutrophils) are the main sources of destructive oxidants that could produce toxic oxygen metabolites through NADPH oxidase. Furthermore, capillary endothelial and alveolar epithelial cells could also generate oxidants *via* similar pathways (Johnson et al., 1981). Under normal physiological conditions, ROS concentrations remain at a moderate rate assisted by the activity of antioxidant enzymes such as superoxide dismutases (SOD) and glutathione peroxidase (GPx) as well as direct antioxidants such as glutathione (GSH) (Zhu et al., 1998). The antioxidant effect has been verified as a major protective mechanism of polysaccharides against lung damage in recent years. Pharmacological studies indicated that CDMP could alleviate H_2_O_2_-induced lung injury through the suppression of MDA and ROS levels *in vitro* (Dong et al., 2020). In addition, oral administration of CDP, DOP, or *Arnebia euchroma* (Royle ex Benth.) I.M.Johnst [Boraginaceae; Arnebiae radix] polysaccharides (AEPs) is beneficial to alleviate lung damage *in vivo* through significantly reducing the levels of MDA and MPO and increasing the levels of SOD in lung homogenates (Ou et al., 2017; Fu et al., 2019; Wen et al., 2021). Likewise, other kinds of plant polysaccharides against oxidative stress injury through the regulation of antioxidant index are listed in Table 1.

#### 2.2.2 Nrf2 pathway

Antioxidant enzymes play an important role in the control of ROS generation, which is usually regulated by nuclear factor erythroid 2-related factor 2 (Nrf2). Nrf2 is regarded as a transcription factor of cellular resistance to oxidative stress. Researchers have revealed that Nrf2-deficient mice have deteriorated pulmonary inflammation and improved susceptibility to hyperoxic lung injury (Reddy et al., 2009). Several studies have demonstrated that the regulation of Nrf2 is a feasible strategy for controlling oxidative stress (Ou et al., 2017). Normally, Nrf2 binds with Kelch-like ECH-associated protein-1 (Keap1) in the cytoplasm. The degradation of Keap1 may prompt Nrf2 to translocate into the nucleus, combine antioxidant responsive elements (AREs), and regulate the expression of antioxidant enzymes containing GPx and HO-1 (hemeoxygenase-1). In addition, AMP-activated protein kinase (AMPK) is a heterotrimeric serine/threonine kinase which plays a unique role in maintaining normal endothelial barrier function and suppressing neutrophil pro-inflammatory activity. Related evidence has proved that the activation of Nrf2 was mediated by AMPK through glycogen synthase kinase 3β (GSK3B) and RAC-alpha serine/threonine-protein kinase (Akt) (Rizvi et al., 2015). Pretreatment with *Lycium barbarum* L [Solanaceae; Lycii fructus] polysaccharide (LBP) or DOP could protect mice against hyperoxia-induced acute lung injury through significantly enhancing Nrf2 in the nucleus and reducing Keap1 in the cytoplasm, with the increase of GPx and HO-1. (Zheng et al., 2019; Wen et al., 2021). Further studies proved that the activation of Nrf2 induced by LBP is mediated by AMPK (Southern et al., 2017; Zheng et al., 2019).

ROS, including superoxide anion radical, hydroxyl radical, and hydrogen peroxide, is usually generated by macrophages and neutrophils (Wang et al., 2020). The role of antioxidants is to intercept the accumulation of these reactive oxygen species. Cytokine storm is noticed in viral infection and causes increased oxidative stress, endothelial cell activation, and neutrophil infiltration. Nrf2 has been deemed as a crucial target for regulating antioxidant response-driven cytoprotective protein expression. However, COVID-19 clinical data are not so numerous. Severe acute respiratory syndrome animal models have proved the impairment of the antioxidant defense system and a lot of reactive oxygen species in the course of a viral infection. Previous studies have shown several polysaccharides against oxidative stress injury by mediating the generation of ROS and activation of Nrf2. Therefore, we assumed that the intervention of antioxidants could become an approach applied in COVID-19 treatment.

#### 2.2.3 MAPK pathway

Accumulating evidence proved that TGF-β1 could not only activate the classical Smad-dependent pathway but also participate in the Smad-independent pathways such as mitogen-activated protein kinase (MAPK), phosphatidylinositide 3-kinase (PI3K), and NF-κB signaling (Derynck and Zhang, 2003). Forefront studies have suggested that redox-sensitive MAPKs are related to ROS generation (Chowdhury et al., 2010). MAPKs belong to a family of structurally related serine/threonine kinases, and extracellular signal-regulated kinase (ERK), c-Jun N-terminal kinase (JNK), and P38 are deemed as the key nodes (Derynck and Zhang, 2003). The activation of MAPKs pathway could be suppressed through decreasing intracellular ROS levels (Ling et al., 2017). In the study of bleomycin (BLM)-induced lung fibrosis, the expression of JNK, ERK, and p38 was obviously inhibited by CDMP, which was given by oral administration. Moreover, CDMP could alleviate H_2_O_2_-induced cellular oxidative stress through the Nrf2/MAPK signaling pathway (Dong et al., 2020).

It is well known that the excitation of inflammatory signaling cascades and the release of pro-inflammatory factors such as TNF-α, IL-6, and IL-1β are pivotal during the development of ALI and the progression of ALI to ARDS. The MAPK families have been shown to be activated by various stimuli, such as LPS and TNF-α, which ultimately result in the production of pro-inflammatory cytokines, chemokines, NO, and ROS. Due to the fact that the MAPK pathway is involved in the synthesis of critical inflammation and oxidative stress mediators, it has been considered as a potential target of polysaccharides in the research and treatment of COVID-19.

### 2.3 Apoptosis

As the terminal stage of lung injury, lung cancer is one of the most common cancers worldwide that is characterized by a rapid proliferation rate and high mortality. The damage caused by cancer is not only limited to the disease itself but also related to the pleural effusion, obstructive pneumonia, and other lung injury-associated complications during the later treatment. Apoptosis plays a significant role in the homeostasis of the human organism. The disruption in the balance between cell proliferation and apoptosis is the leading cause of pulmonary tumor development and progression (Wong, 2011). Promoting apoptosis of lung cancer cells is regarded as a promising therapeutic measure.

#### 2.3.1 Cell cycle

The cell cycle is a highly regulated process that consists of two distinct phases. The mitotic phase (M), where a cell undergoes cell division and interphase, can be further divided into G1 (pre-DNA synthesis), S (DNA synthesis), and G2 (pre-division) phases (Norbury and Nurse, 1992). Another G0 phase (quiescence) refers to cells not involved in cell cycle but have the ability to divide. As a general rule, cells can turn into G1 from the quiescent state through mitogenic stimuli. The G1 phase is regarded as the initiation step in cell cycle progression. In the S phase, DNA is synthesized by cells, with a content of between 2N and 4N. Following this phase, cells enter the G2 phase to prepare for the M phase and divide into two independent daughter cells. Tumor proliferation capacity could be evaluated through the expression of proliferating cell nuclear antigen (PCNA). The synthesis of PCNA is involved in the cell proliferation of lung cancer injury (Gu et al., 2016). The level of PCNA was increased in the G1 and S phases. In addition, this antigen could not be detected in quiescent cells.

The lung-protective effect of *Glehnia littoralis* (A.Gray) F.Schmidt ex Miq [Apiaceae; Glehniae radix] polysaccharide (GLP) in A549 cells has been reported to interfere with the apoptosis pathway. Following treatment with GLP, the expression of PCNA was significantly downregulated. Moreover, flow cytometry detection revealed that GLP could arrest the cell cycle in the S and G2/M phases compared with the control group in a dose-dependent way (Wu et al., 2018). Additionally, cyclin B1 is synthesized in the late and early stages of the S and G2 phases, respectively. It could be degraded before the completion of mitosis and thus played an important role in the S phase block (Murray et al., 1989). Pretreatment with a pectic polysaccharide isolated from *Houttuynia cordata* Thunb. showed that A549 cells were significantly arrested in the S and G2 phases. Moreover, further analysis verified that the expression of cyclin B1 was markedly increased in a dose-dependent manner (Han et al., 2018).

The latest research showed a novel pathogenic mechanism, the overexpression of T-cell surface Fas and DNA damage attributed to AngII-driven ROS generation by the monocytes in some COVID-19 patients, leading to peripheral mononuclear blood cell apoptosis (Kundura et al., 2022). It is worth noting that intensive care unit (ICU) patients present more T-cell apoptosis and lymphopenia than non-ICU; nevertheless, the plasma level of AngII and monocytic ROS production are lower. ROS-induced programmed cell death may result in an immune deficiency. Unfortunately, the correlation between the cell cycle and COVID-19 has not been elucidated. According to theoretical analysis, polysaccharides may benefit the cell cycle through regulating the generation of ROS.

#### 2.3.2 PI3K/Akt pathway

Accumulating research studies have shown that the Akt signaling pathway regulates various downstream targets mediating tumor-associated cell processes including cell growth, cell cycle progression, migration, and angiogenesis (Cheng et al., 2005). PI3K is a major upstream node of Akt, which has the ability to convert phosphatidylinositol-4, 5-bisphosphate into phosphatidylinositol-3, 4, 5-triphosphate. A recent study has shown that the downregulation of the PI3K/Akt pathway could induce cell apoptosis and inhibit cell proliferation of the A549 human non-small lung cancer cell line through significantly increasing the mRNA expression of caspase-3, fatty acid synthase (Fas), Fas antigen ligand (FasL), and apoptosis regulator BAX (Bax) and decreasing the mRNA expression of B-cell lymphoma 2 (Bcl-2) (Qu et al., 2018).

The COVID-19-induced cytokine storm results in micro- and macrovascular thrombosis formation. A related study has been shown that COVID-19 deaths are obviously related to higher d-dimer and fibrin degradation product levels compared to survivors (Abu-Eid and Ward, 2021). Due to the role of the PI3K/AKT pathway in platelet activation (Chen et al., 2019), targeting this pathway has the potential to prevent the formation of thrombosis (Sekhawat et al., 2020). Furthermore, PI3K plays an important role in leucocyte extravasation in the ALI model (Kral-Pointner et al., 2019). In addition to anticoagulants, the application of plant polysaccharides is considered as a therapeutic approach for intravascular coagulation associated with COVID-19.

#### 2.3.3 Caspase cascade pathway

The caspases are prominent among the death proteases, which belong to a family of cysteine-dependent aspartate-directed proteases. Related studies have revealed that activation of a few caspase families is associated with programmed cell death (Philchenkov, 2004). In particular, the stimulation of caspase-9/3 is treated as a crucial target for the propagation of the apoptotic signal, which could activate downstream caspases and trigger apoptosis (Ho and Hawkins 2005). Moreover, the increase of cytochrome c from the mitochondria into the cytosol is one of the prominent events in this approach (Balk et al., 1999). However, the release of cytochrome c was regulated by Bcl-2 family proteins including the anti-apoptotic and pro-apoptotic proteins Bcl-2 and Bax (Kluck et al., 1997). Bcl-2 has the ability to obstruct cytochrome c efflux *via* attaching to the outer mitochondrial membrane (Terrones et al., 2004). In contrast, Bax could be transferred to the outer mitochondrial membrane from the cytoplasm, which contributes to cytochrome c release (van Loo et al., 2002).

Various pieces of evidence demonstrated that the induction of lung cancer cells contributed to alleviating the progress of lung injury. For example, polysaccharide derived from *Scleromitrion diffusum* (Willd.) R.J.Wang [Rubiaceae; Hedyotidis diffusae herba] (SDP) could induct the release of cytochrome c from the mitochondria into the cytosol prior to the stimulation of caspase-9 and -3 in A549 cells (Lin et al., 2019). *Echinacea purpurea* (L.) Moench [Asteraceae; Purple coneflower herb] polysaccharide (EPP) that was administrated significantly alleviated LPS-induced cell apoptosis through increasing and decreasing the Bax and Bcl-2 expression in lung tissues in a dose-dependent way, respectively (Zhang et al., 2020)*.* Moreover, the anti-apoptosis effect of polysaccharide from *Coix lacryma-jobi var. ma-yuen* (Rom.Caill.) Stapf [Poaceae; Coicis Semen] (CLP) on the human non–small-cell lung cancer A549 cell line by disrupting the mitochondrial membrane potential, which further results in the activation of caspase-3 and caspase-9, has also been reported (Lu et al., 2013).

The infection of COVID-19 could affect multiple systems including programmed cell death. Maleki et al. proved that the expression of caspase-3 is higher in the seminal plasma of COVID-19 patients than in the healthy control (Hajizadeh Maleki and Tartibian, 2021). According to the latest studies, plasma-derived extracellular vesicles stimulated caspase-3 activity and reduced the rate of survival in human pulmonary microvascular endothelial cells of severe COVID-19 patients (Krishnamachary et al., 2020; Magro et al., 2021). The level of caspase-3 may be affected by coagulation irregularity, organ damage, and inflammation. Drugs that regulate caspase-3 expression should be given more attention in the treatment of COVID-19 patients.

### 2.4 Autophagy

#### 2.4.1 PAK1/Akt/mTOR signaling

Autophagy is an evolutionally conserved process in which cytoplasmic contents (e.g., proteins, lipids, and nucleic acids) and damaged organelles (e.g., mitochondria and peroxisomes) are sequestered by the double-membrane structure and delivered to the lysosomes for degradation (Marx, 2015). In general, autophagy is particularly regarded as a cytoprotective mechanism. Beclin1 and Atg5 are two autophagy-related proteins that are involved in the formation of autophagosomes. Related research suggests that *Ipomoea batatas* (L.) Lam. [Convolvulaceae; *Ipomoea* radix] polysaccharide (IBP) could promote autophagosome formation *via* activating the expression of Beclin1 and Atg5 (Bu et al., 2020). Moreover, treatment with IBP in lung cancer cells may enhance autophagy by decreasing the interaction of Beclin1/Bcl-2. In addition, the formation of the Beclin1–PI3K complex was also inhibited, which probably contributes to accelerating autophagy through the Akt/mTOR signaling pathway (Bu et al., 2020). Furthermore, p21-activated kinase (PAK1) is associated with cell proliferation, invasion, and usually aberrant expression in various tumors (Higuchi et al., 2008). Bu et al. (2020) first discovered that IBP is a potential medicine against lung cancer growth through inducing PAK1/Akt/mTOR-mediated autophagy.

PAK1 is an important “pathogenic” kinase whose abnormal activation is responsible for various disorders including cancers, HIV, and COVID-19. Recently, it has been found that PAK1 can be suppressed by a tumor-suppressing phosphatase, and it has been shown to inactivate the coronavirus-induced lung inflammation and fibrosis (Lu et al., 2020). The ACE2-mediated PAK1 signaling pathway could regulate the expression of related genes (Chen et al., 2010). Thus, PAK1-blockers, interfering with the pathogenic process, could act as potential therapeutic agents for COVID-19 patients.

### 2.5 Epithelial–mesenchymal transition

Epithelial–mesenchymal transition (EMT) has been perceived as the terminal phase in various chronic pathology damages, especially in lung and kidney tissue. It is usually associated with cell conjunction, migration, invasion, and fibroblast-like morphology changes (Shi et al., 2020). EMT is characterized as the alteration of morphology, with the mesenchymal cells presenting a spindle shape with tentacles. The regulation of epithelial-related biomarkers (claudin-1 and E-cadherin) and mesenchymal-related targets (vimentin and N-cadherin) has been regarded as pivotal biomarkers in the process of EMT (Kalluri and Weinberg, 2009). In addition, pulmonary interstitial fibrosis is a hallmark of lung injury that is characterized by the excessive production and deposition of extracellular matrix (ECM) and the increase of EMT. Even more remarkably, matrix metalloproteinases (MMPs) are essential proteolytic enzymes that degrade ECM (Nelson et al., 2000). The regulation of MMPs may be beneficial to lung injury. However, the activity of MMPs could be negatively regulated by tissue inhibitors of metalloproteinase (TIMPs) and thus protect the basement membrane from proteolysis (Lambert et al., 2004). Respiratory symptoms are the main manifestation of COVID-19; the MMPs play essential roles in the lung physiology against lung injury. A recent study has elucidated a significant relation between MMP levels and COVID-19 in more detail (Avila-Mesquita et al., 2021). The levels of MMP-2 and MMP-9 could forecast the risk of in-hospital death, suggesting potential pathophysiologic and prognostic effects (Avila-Mesquita et al., 2021).

#### 2.5.1 EGFR/Akt/ERK pathway

It is well known that the overexpression of cyclooxygenase-2 (COX-2) may accelerate EMT progression and invasion. More importantly, the expression of epidermal growth factor receptor (EGFR) could be represented as a negative feedback that is controlled by COX-2 in patients suffering from lung cancer by mediating the activation of several signaling cascades (Prado et al., 2010). As the downstream targets of EGFR, the PI3K/Akt and MAPK signaling pathways also play indispensable roles in tumor initiation and development (Gao et al., 2013). Furthermore, a great number of evidence suggests that the PI3K/Akt and MAPK pathways are always involved in the mediation of EMT from various aspects (Westermarck and Kähäri, 1999; Bae et al., 2006). SDP significantly restrained the cell adhesion, invasion, and migration of A549 cells in a dose-dependent manner *via* reducing the expression of MMP-2 and MMP-9 and increasing the expression of TIMP-2 and TIMP-9. Meanwhile, treatment with SDP could effectively downregulate the expression of N-cadherin and vimentin and upregulate E-cadherin expression, which is related to inactivating the EGFR/Akt/ERK pathways and repressing COX-2 protein expression (Linet al., 2019).

#### 2.5.2 TGF-β1/SMAD pathway

In previous studies, TGF-β1 plays a prominent role in the activation of EMT-induced transcription factors of several signaling initiators (EGF) (Gonzalez and Medici, 2014). EMT and metastasis may be mediated by TGF-β1 through the non-canonical and/or canonical pathways. Under circumstances of the non-canonical signaling pathways, the ERK and p38 MAPK signaling pathways could be activated *via* the stimulation of TGF-β1 that would probably be seen as a result of the dual specific kinase activity of the TGF-β1 receptor (Derynck and Zhang, 2003). The augmented TGF-β1-induced transcription by ERK signaling could not only restrain the expression of E-cadherin and GSK3 but also stimulate the expression of N-cadherin and MMPs. Simultaneously, the activation of p38 MAPK and JNK may be caused by the integration of the ubiquitin ligase TRAF6 and TGF-β1 receptors, and then the upstream kinase TAK1 will be motivated. Nevertheless, the successful completion of signal transduction processes requires the cooperation of SMAD2/3 (Sorrentino et al., 2008).

Lim et al. (2020) also demonstrated that polysaccharides derived from *Diospyros kaki* L. f. [Ebenaceae; Kaki calyx] (DKB) could significantly inhibit lung tumor cells by increasing expression of E-cadherin and declining expression of N-cadherin and vimentin, and then the levels of MMP-2 and MMP-9 could be suppressed with cleaving PARP, while the SMAD2/3 and ERK/p38 signaling pathways could be activated finally. In the study of BLM-induced early stage of lung injury, the expression intensity of TGFβ1, Smad2/3, and pSmad2/3 were dramatically increased by DOP, which was given by oral administration. In addition, the expression of α-SMA could be attenuated with lessened collagen deposition. In general, DOP exerted lung protective effects by blocking activation of the TGFβ1-Smad2/3 axis (Chen et al., 2018).

Depending on the different cell types and microenvironment, Smad signaling can be stimulated through the activation of type 1 and 2 receptors by TGF-β1. Inappropriate activation of the TGF-β1 pathway will lead to the increased generation of profibrotic mediators and ECM proteins. Zhou et al. (2012) revealed that pulmonary overexpression of TGF-β1 substantially promotes the activation of EGFR signaling, which is foundational for TGF-β/SMAD signaling. In addition, fibroblast proliferation could be stimulated *via* EGFR signaling by activation of PI3K/AKT and MAPK/Erk. At the beginning of COVID-19 infection, the mRNA expression of TGF-β1 in upper airway samples is higher than in controls (Montalvo Villalba et al., 2020). Polysaccharide extracts from plants could act on several core targets, which may contribute to the treatment of COVID-19 infection.

#### 2.5.3 FoxO signaling

Forkhead box (FoxO) transcription factors are related to the cellular responses to bacterial stimuli and are regarded as important targets of innate immune functions in respiratory epithelial cells. Previous researchers have proved that FoxO signaling is indispensable to resisting highly stressful conditions. Moreover, the activation of FoxO3 has been detected in airway epithelial cells of lung injury-related disease (Seiler et al., 2013). Interestingly, the differentiation antagonizing non-protein coding RNA (DANCR) is also known as a long noncoding RNA (lncRNA) (Kretz et al., 2012). It has been proved that DANCR could promote EMT progression and invasion capability of malignant cells and play a special role in cell proliferation, migration, invasion, and stem cell differentiation (Jia et al., 2016). In the case of DANCR activation, an adaptor protein AU-binding factor 1 (AUF1) may be related to mediating EMT and fibrogenesis through increasing FoxO3 protein expression. Pretreatment with *Angelica sinensis* (Oliv.) Diels [Apiaceae; Angelicae sinensis radix] (ASP) protected mice against BLM-triggered higher collagen deposition through significantly reducing collagen-1 protein levels *in vivo*. In addition, the expression of lncRNA DANCR could also be downregulated by ASP and then lead to the inactivation of FoxO3 translation in an AUF1-dependent manner *in vitro* and *in vivo* (Qian et al., 2020).

So far, the relationship between FoxO signaling and COVID-19 has not been reported. Plant polysaccharides against lung injury *via* the FoxO3 target have been studied. In the not-too-distant future, the potential mechanism for the treatment of COVID-19 patients with polysaccharides through the FoxO target will make a breakthrough.

### 2.6 Immunomodulatory

With COVID-19 ravaging around the world, it has been recognized that effective body immunity plays an important part in resisting viruses and promotes prognosis of disease. Immune cells such as lymphocytes, NK cells, macrophages, neutrophils, and monocytes are the main targets of coupling between specific proteins and immunostimulatory polysaccharides. Therefore, polysaccharides with immunoregulatory activity can directly or indirectly interact with the body’s immune system and initiate a series of molecular interactions to protect against lung injury.

#### 2.6.1 Complement system

The complement system consists of more than 30 plasma and membrane-bound proteins and is regarded as a non-specific host immune response. It could provoke immediately upon injury and reach a greater level during resuscitation (Fruchterman et al., 1998). However, previous studies have demonstrated that the inappropriate activation of the complement system always associated with pathogenesis in tissue injuries (Mulligan et al., 1996; Morgan and Harris, 2003). In the progress of complement activation, C3a, C4a, and C5a were generated from the cleavage of the C3, C4, and C5 that may cause the degranulation of endothelial cells, mast cells, or phagocytes, resulting in inflammatory responses and fatal shock-like syndrome (Ember and Hugli, 1997). The regulation of complement activity could be supposed to be an initial step in lung injury *via* the classical and alternative pathways (Langlois and Gawryl, 1988; Mulligan et al., 1996). The destruction of endothelial integrity could induce injured pulmonary cells to release some tissue factors or enzymes, which may result in the activation of the complement cascade during the lung injury process (Gralinski et al., 2018). Experiments revealed that HCP and AEP exhibited anti-complementary activity through both classical and alternative pathways by obstructing the crucial components C3 and C4 *in vitro* and *in vivo* (Ou et al., 2017; Lu et al., 2018). Moreover, pretreatment with HCP protected rats against ALI-induced hemorrhagic shock plus LPS through the reduction of serum complement levels in the rats (Lu et al., 2018). Interestingly, the blockade of C5a has been demonstrated to alleviate the increasing level of TNF-α and mitigate chemotactic and phagocytotic dysfunctions of neutrophils in trauma-induced ALI (Flierl et al., 2008). Similarly, HCP could significantly palliate inflammation in the mice model *via* decreasing the influx of inflammatory cells that are mediated by complement split products (C5a and C3d) (Xu et al., 2015).

In addition, the degree of complement activation could be reflected by the content of C3c, which was one of the stable pyrolyzed products during this process (Palarasah et al., 2010). As shown in previous research, pretreatment with CDP protected mice against H1N1 virus-infected acute lung injury by notably decreasing the level of C3c deposition in lung tissue (Fu et al., 2019), which demonstrates an inhibition of excessive activation in the complement system.

Complement activation correlated well with the severity of disease in viral pneumonia, respiratory distress syndrome, and multi-organ failure. As for COVID-19, the complement system could be activated in lung epithelial cells, driven by the transcription of factor B and C3a (Yan et al., 2021). Additionally, several products of the complement system, including C5a, C4a, and C3b, were detected in the sera of patients with COVID-19 (Holter et al., 2020). Therefore, the application of plant polysaccharides as an effective therapeutic strategy should be investigated in the future through restraining the excitation of complement pathways.

#### 2.6.2 Lymphocyte

The immune system plays a significant role in lung injury defense. The spleen acts as an important organ because of maturing lymphocytes that have been directly involved in immune response by releasing cytokines. T and B lymphocytes are mainly responsible for cellular immunity and humoral immunity, respectively. Various evidence have proven that NK cells are attributable to a type of non-specific lymphocyte (Ravetch and Lanier, 2000; Moretta et al., 2001). *Panax ginseng* C.A.Mey. [Araliaceae; Ginseng radix et rhizoma] (PGP) given orally significantly improves the killing activity of NK cells from the splenocytes and the Lewis lung carcinoma-bearing mouse model (Wang et al., 2015).

As we all know, CD4^+^ and CD8^+^ are T-helper (Th) and T-cytotoxic (Tc) lymphocytes, respectively. A numbers of investigators have concluded that the high ratio of CD4^+^/CD8^+^ may be conducive to alleviating tumor injury (Shen et el., 2013). Wang et al. (2015) showed that the ratio of CD4^+^/CD8^+^ was increased after intragastric administration of PGP in peripheral blood. More recently, chemokines and their receptors that act as potential targets for immunomodulation have played a key role in the immune system (Hill and Sarvetnick, 2002). During this process, CCR6 and its chemokine ligand CCL20 have emerged for their potential role in the regulation of humoral immune responses at the cellular level. Shi et al. (2020) demonstrated that oral administration of HCP could first restore the balance of Th17/Treg cells in the lung tissue and then induce the CCL20 downregulation and mediate the balance of Th17/Treg cells carrying CCR6. In addition, Th1 and Th2 are the two major subtypes of CD4^+^ T lymphocytes. Th1 usually inhibits tumor cell proliferation with the presence of IL-2, IFN-γ, and IL-12 in cell-mediated immunity. Th2 mediates the humoral immune and could generate IL-4, IL-5, and other cytokines and then accelerate tumor cell proliferation (Hong et al., 2008). Under normal circumstances, Th1/Th2 cells stay in a relative balance status. Nevertheless, the dynamic balance of Th1/Th2 cytokines could be changed due to abnormal immune function in patients (Chechlińska ete al., 2004). Ma et al. (2014) also reported that administration of PGP could improve and decrease the levels of Th1 (INF-γ and IL-2) and Th2 cytokines (IL-4 and IL-5) and thus increase the ratio of Th1/Th2 cytokines (INF-γ/IL-4 and IL-2/L-5) in clinical patients.

In coronavirus family infected patients, the declension of lymphocytes is detected (Al-Tawfiq et al., 2017). The potential mechanism may be related to the direct attack of the virus and/or immune-mediated apoptosis of lymphocytes. In addition, the cells that exhibit ACE2 receptors are susceptible to being infected by COVID-19 (Xu et al., 2020). The ACE2 receptors are likely the cell receptors of COVID-19 and a receptor for coronavirus. When the complement system is excessively activated, lung injury will be aggravated. The immunomodulatory activity, one of the most significant properties of plant polysaccharides, plays a significant part in lung protection.

## 3 Future prospect

COVID-19 has developed into the source of a global pandemic. The greatest challenges for the world and China are to contain its spread. Chinese medicinal plants with thousands of years of practice in the prevention of infectious diseases may play an important role in this COVID-19 pandemic. During the past decades, a number of polysaccharides with lung-protection properties have been extracted from plants, and their potential for therapeutic applications has attracted considerable attention of scientists to work on them all over the world. Plant polysaccharides with specific structures or certain molecular weights exhibit different activities. However, the structure–activity relationships of polysaccharides are not yet entirely understood. Metabolism and excretion of plant polysaccharides should be given more attention because liver or kidney damage may be induced by the unreasonable metabolic pathway.

Moreover, different kinds of plant polysaccharides could regulate the gut microbiota and play important roles in a variety of physiologic or pathologic events. Through clinical observation, COVID-19 patients have intestinal flora disorders and barrier function damage, which are regarded as the turning points in the prognosis of viral infection. The application of the exterior of the lung and large intestine, the basic theory of TCM clinical treatment of COVID-19, could achieve satisfactory effects. Therefore, research on maintaining the gut microbiota balance will provide new targets and ideas for the treatment of COVID-19.

According to the theory of TCM in tropism of taste, meridian distribution, a part of Chinese medicine property, is one of the foundations for clinical prescription. According to the present studies, *Houttuynia cordata Thunb.* (Yu Xing Cao) and *Astragalus* (Huang Qi) are distributed in lung channel to alleviate lung injury. Other plants that belong to the lung meridian should also be considered as a condition of polysaccharide against lung injury in future investigation.

## 4 Conclusion

The fields of pulmonary airway, vascular, interstitial tissue, and even lung cancer injury have attracted more attention in order to find pulmonary protective treatments for COVID-19 patients. As a promising source in the treatment of lung diseases, the lung-protective activity of polysaccharides has been demonstrated by studies using *in vitro* and *in vivo* models. In the present review, the physiological activities and underlying mechanisms of 19 kinds of plant polysaccharides were systematically summarized and analyzed. Moreover, novel insights for future research have also been preliminary provided, which lays the foundation for the further research and development of polysaccharides. Therefore, plant polysaccharides may be considered as a safe and effective alternative therapy for pulmonary injury in COVID-19 patients.

## Glossary

SARS: severe acute respiratory syndrome
MERS: Middle East respiratory syndrome
SARS-CoV-2: severe acute respiratory syndrome–coronavirus 2
COVID-19: coronavirus disease 2019
ALI: acute lung injury
ARDS: acute respiratory distress syndrome
ACE2: angiotensin-converting enzyme II
MUC5AC: mucin-5AC
FEV1% pred: forced expiratory volume in 1 s percent predicted
COPD: chronic obstructive pulmonary disease
AQPs: aquaporins
DOP: *Dendrobium officinale* Kimura and Migo polysaccharide
IL-6: interleukin 6
CRP: C-reactive protein
AMP: *Astragalus mongholicus* Bunge polysaccharide
OVA-LPS: ovalbumin-lipopolysaccharide
IL-1β: interleukin-1 beta
TNF-α: tumor necrosis factor
IL-8: interleukin 8
BALF: bronchoalveolar lavage fluid
TLR: toll-like receptor
NF-κB: nuclear transcription factor-κB
LPS: lipopolysaccharide
CDP: *Cedrus deodara* (Roxb. ex D.Don) G.Don polysaccharide
TIR: Toll/IL-1 receptor
NF-κB: nuclear transcription factor-κB
MYD88: myeloid differentiation primary response gene 88
IRAK-4: IL-1 receptor-associated kinase-4
IKK: inhibitor of NF-kappaB kinase
IκB: inhibitor of NF-κB
NTP: *Nitraria tangutorum* Bobrov polysaccharide
CDMP: *Cedrus deodara* (Roxb. ex D.Don) G.Don polysaccharide
HCP: *Houttuynia cordata* Thunb. polysaccharide
ER: endoplasmic reticulum
UPR: unfolded protein response
PERK: protein kinase RNA-like ER kinase
IRE1: inositol-requiring enzyme 1
ATF6: activating transcription factor 6
BIP: heavy-chain binding protein
CHOP: C/EBP homologous protein
BCP: *Bupleurum chinense* DC. polysaccharide
ROS: reactive oxygen species
NSPs: neutrophil serine proteases
NETs: neutrophil extracellular traps
HNE: human neutrophil elastase
IFN-γ: interferon-γ
MCP-1: monocyte chemoattractant protein-1
BSSP: *Bassia scoparia* (L.) *A.J.Scott* polysaccharides
NADPH: nicotinamide adenine dinucleotide phosphate
TGF-β: transforming growth factor beta
SOD: superoxide dismutase
GPx: glutathione peroxidase
GSH: glutathione
AEP: *Arnebia euchroma* (Royle ex Benth.) I.M.Johnst. polysaccharide
Nrf2: nuclear factor erythroid 2-related factor 2
Keap1: Kelch-like ECH-associated protein-1
AREs: antioxidant responsive elements
HO-1: hemeoxygenase -1
AMPK: AMP-activated protein kinase
GSK3B: glycogen synthase kinase 3β
Akt: RAC-alpha serine/threonine-protein kinase
LBP: *Lycium barbarum* L. polysaccharide
MAPK: mitogen-activated protein kinase
PI3K: phosphatidylinositide 3-kinase
ERK: extracellular signal-regulated kinase
JNKs: c-Jun N-terminal kinases
BLM: bleomycin
PCNA: proliferating cell nuclear antigen
GLP: *Glehnia littoralis* (A.Gray) F.Schmidt ex Miq. polysaccharide
Bcl-2: B-cell lymphoma 2
Fas: fatty acid synthase
FasL: Fas antigen ligand
EPP: *Echinacea purpurea* (L.) Moench polysaccharide
CLP: *Coix lacryma-jobi var. ma-yuen* (Rom.Caill.) Stapf polysaccharide
IBP: *Ipomoea batatas* (L.) Lam. polysaccharide
PAK1: p21-activated kinase
PTEN: tumor-suppressing phosphatase
EMT: epithelial–mesenchymal transition
ECM: extracellular matrix
MMPs: metalloproteinases
TIMPs: tissue inhibitors of metalloproteinase
EGFR: epidermal growth factor receptor
SDP: *Scleromitrion diffusum* (Willd.) R.J.Wang polysaccharide
DKP: *Diospyros kaki* L.f. polysaccharide
DANCR: differentiation antagonizing non-protein coding RNA
AUF1: AU-binding factor 1
PGP: *Panax ginseng* C.A.Mey. polysaccharide.
